# Positive Effects of NPY1 Receptor Activation on Islet Structure Are Driven by Pancreatic Alpha- and Beta-Cell Transdifferentiation in Diabetic Mice

**DOI:** 10.3389/fendo.2021.633625

**Published:** 2021-02-25

**Authors:** Ryan A. Lafferty, Neil Tanday, R. Charlotte Moffett, Frank Reimann, Fiona M. Gribble, Peter R. Flatt, Nigel Irwin

**Affiliations:** ^1^ SAAD Centre for Pharmacy and Diabetes, Ulster University, Coleraine, United Kingdom; ^2^ Wellcome Trust-MRC Institute of Metabolic Science, University of Cambridge, Cambridge, United Kingdom

**Keywords:** peptide YY, sea lamprey PYY, diabetes, transdifferentiation, streptozotocin

## Abstract

Enzymatically stable and specific neuropeptide Y1 receptor (NPYR1) agonists, such as sea lamprey PYY(1-36) (SL-PYY(1-36)), are believed to improve glucose regulation in diabetes by targeting pancreatic islets. In this study, streptozotocin (STZ) diabetic transgenic *Glu^CreERT2^*;*ROSA26-eYFP* and *Ins1* ^Cre/+^;*Rosa26-eYFP* mouse models have been used to study effects of sustained NPYR1 activation on islet cell composition and alpha- and beta-cell lineage transitioning. STZ induced a particularly severe form of diabetes in *Ins1* ^Cre/+^;*Rosa26-eYFP* mice, but twice-daily administration (25 nmol/kg) of SL-PYY(1-36) for 11 days consistently improved metabolic status. Blood glucose was decreased (p < 0.05 - p < 0.001) and both fasted plasma and pancreatic insulin significantly increased by SL-PYY(1-36). In both *Glu^CreERT2^*;*ROSA26-eYFP* and *Ins1* ^Cre/+^; *Rosa26-eYFP* mice, STZ provoked characteristic losses (p < 0.05 - p < 0.001) of islet numbers, beta-cell and pancreatic islet areas together with increases in area and central islet location of alpha-cells. With exception of alpha-cell area, these morphological changes were fully, or partially, returned to non-diabetic control levels by SL-PYY(1-36). Interestingly, STZ apparently triggered decreased (p < 0.001) alpha- to beta-cell transition in *Glu^CreERT2^*;*ROSA26-eYFP* mice, together with increased loss of beta-cell identity in *Ins1* ^Cre/+^;*Rosa26-eYFP* mice, but both effects were significantly (p < 0.001) reversed by SL-PYY(1-36). SL-PYY(1-36) also apparently reduced (p < 0.05) beta- to alpha-cell conversion in *Ins1* ^Cre/+^;*Rosa26-eYFP* mice and glucagon expressing alpha-cells in *Glu^CreERT2^*;*ROSA26-eYFP* mice. These data indicate that islet benefits of prolonged NPY1R activation, and especially restoration of beta-cell mass, are observed irrespective of diabetes status, being linked to cell lineage alterations including transdifferentiation of alpha- to beta-cells.

## Introduction

Recent investigations have confirmed beneficial effects of sustained activation of pancreatic beta-cell neuropeptide Y1 receptors (NPY1R’s) on the growth, survival and overall secretory function of insulin-producing cells (1–5). In this regard, enzymatically stable PYY(1–36) peptides from phylogenetically ancient fish have been demonstrated to function as long-acting, potent and specific NPY1R agonists (4). Although the NPY1R is not believed to be expressed on pancreatic alpha-cells, PYY is known to be locally secreted from these alpha-cells (6), and positive effects of the piscine-derived PYY(1-36) peptides on alpha-cell morphology and glucagon secretion have also been noted (4). Since diabetes is a metabolic disorder characterized by both aberrant alpha- and beta-cell secretory function (7, 8), it suggests NPY1R modulation may have extremely credible antidiabetic potential (9). Indeed, the most efficacious piscine-derived PYY(1-36) sequence, namely sea lamprey PYY(1-36) (SL-PYY(1-36)) (4), has already been shown to combat beta-cell loss in diabetic rodents *via* augmentation of beta-cell proliferation and a reduction in apoptosis (4).

These positive islet effects are unlikely to account for the full pancreatic architectural benefits of SL-PYY(1-36). As such, although changes in beta-cell growth and survival were significantly improved following sustained SL-PYY(1-36) administration, relative changes were modest in quantitative terms, with less obvious impact on alpha-cell turnover (4). In relation to this, recent elegant cell lineage tracing studies have highlighted the transitioning ability of mature islet alpha- and beta-cells, leading to alterations in islet architecture, especially in diabetes (10–13). Thus, despite their conflicting roles in the control of blood glucose, alpha- and beta-cells share similar transcriptomes (14, 15), and it seems plausible that SL-PYY(1-36) might influence transdifferentiation of both cell types, to help elicit the observed NPY1R-mediated improvements in pancreatic islet morphology (4, 5, 16). Importantly in terms of clinical relevance, islet cell transdifferentiation is not restricted to rodents and has been evidenced in human beta-cells (17–20) and islet cells from patients with diabetes (21). Therefore, the present study was conducted using transgenic mice with alpha- and beta-cell lineage tracing capabilities, to investigate the contribution of transdifferentiation of alpha- and beta-cells to SL-PYY(1-36)-induced improvements of pancreatic islet architecture in diabetes.

Fully characterized *Glu^CreERT2^*;*ROSA26-eYFP* and *Ins1* ^Cre/+^;*Rosa26-eYFP* transgenic mouse models (22, 23) were used to directly investigate the impact of sustained NPY1R signaling on both alpha- to beta-, as well as beta- to alpha-cell, transdifferentiation, respectively. It is generally considered that islet cell transdifferentiation only occurs following severe pancreatic insult, as experienced in diabetes (11). Thus, in the current setting chemically induced beta-cell ablation was used to provoke and study transdifferentiation of individual alpha- and beta-cells in *Glu^CreERT2^*;*ROSA26-eYFP* and *Ins1* ^Cre/+^;*Rosa26-eYFP* mice (10). Importantly, this method of diabetes induction aligns well with previous investigations on the antidiabetic and pancreatic architectural benefits of SL-PYY(1-36) treatment (4). Therefore, the impact of sub-chronic pharmacological upregulation of NPY1R pathways in the two diabetic transgenic rodent models was investigated, through 11-day twice daily administration of SL-PYY(1-36). Taken together, our datasets suggest that pancreatic islet architectural benefits of prolonged NPY1R activation in diabetes are partly linked to positive alteration of the transdifferentiation of both alpha- and beta-cells.

## Materials and Methods

### Peptides

SL-PYY(1-36) was supplied by Synpeptide Ltd (Shanghai, China) at greater than 95% purity and characterized in-house by HPLC with MALDI-TOF, as described previously (24). Specificity of SL-PYY(1-36) for NPY1R has previously been confirmed using receptor knockout cell lines (4).

### Generation of Glu^CreERT2^;Rosa26-eYFP and Ins1^Cre/+^;Rosa26-eYFP mice

Transgenic *Glu^CreERT2^*;*Rosa26-eYFP* and *Ins1^Cre/+^*;*Rosa26-eYFP* C57BL/6 mice were bred in-house at the Biomedical and Behavioural Research Unit (BBRU) at Ulster University, Coleraine. Full details of the generation and characterization of mouse models are provided by Campbell et al., 2020 (22) and Tanday et al., 2020a (23), respectively. It is important to note that *Glu^CreERT2^* and *Ins1^Cre/+^* are not equivalent lineage tracers. As such, *Ins1^Cre/+^* will come on as soon as a cell makes *Ins1*, whereas *Glu^CreERT2^* will only label cells producing glucagon when concomitantly exposed to tamoxifen. PCR genotyping for each colony was employed as previously described (12, 22). *Glu^CreERT2^*;*Rosa26-eYFP* animals were administered tamoxifen (7 mg/mouse bw, i.p.), 2 days prior to the first STZ injection, to induce expression of the alpha-cell fluorescent lineage marker protein. All experiments were carried out under the UK Animals (Scientific Procedures) Act 1986 & EU Directive 2010/63EU. Animals were used at 14 weeks of age and were maintained in an environmentally controlled unit at 22 ± 2°C with a 12 h dark and light cycle and given *ad libitum* access to standard rodent diet (10% fat, 30% protein and 60% carbohydrate; Trouw Nutrition, Northwich, UK) and drinking water.

### Experimental Protocols

Multiple low dose streptozotocin (STZ) injection regimen (4 h fast, 50 mg/kg bw, i.p., in sodium citrate buffer, pH 4.5) was employed in both mouse models for 5 consecutive days to induce diabetes. Upon biochemical confirmation of diabetes development, mice received twice-daily (09:00 and 17:00 h) treatment with either saline vehicle (0.9% (w/v) NaCl) or SL-PYY(1-36) (25  nmol/kg bw) for 11 days. This dosing regimen was based on previous studies with SL-PYY(1-36) and related peptides in STZ-diabetic mice (4, 5). Body weight, blood glucose, cumulative food and fluid intake as well as circulating glucose levels were assessed at regular intervals. At the end of the treatment period, non-fasting and fasting plasma insulin, as well as non-fasting plasma glucagon concentrations were determined. At termination, pancreatic tissues were excised, divided longitudinally, and processed for either determination of pancreatic hormone content following acid/ethanol protein extraction or fixed in 4% PFA for 48 h at 4°C for histological analysis (4, 5). Despite using an identical approach to STZ administration in both groups of transgenic mice, *Ins1^Cre/+^*;*Rosa26-eYFP* mice, derived directly from breeding pairs obtained from Jackson Laboratories (Bar Harbor, Maine, USA), developed a more severe diabetes phenotype. This most likely reflects differences in background genome compared to the *Glu^CreERT2^*;*Rosa26-eYFP* mouse model, which affected STZ susceptibility (25, 26).

### Immunohistochemistry

Fixed tissues were processed and embedded in paraffin wax blocks using an automated tissue processor (Leica TP1020, Leica Microsystems), and 5 μm sections cut on a microtome (Shandon Finesse 325, Thermo Scientific), and sections selected at intervals of every 10 sections. Slides were dewaxed by immersion in xylene and rehydrated through a series of ethanol solutions of reducing concentration (100%–50%). Heat-mediated antigen retrieval was then carried out in citrate buffer. Sections were blocked in 4% BSA solution before 4°C overnight incubation with appropriate primary antibodies (**Table 1**), Slides were then rinsed in PBS and incubated for 45 min at 37°C with appropriate Alexa Fluor secondary antibodies (**Table 1**). Slides were finally incubated with DAPI for 15 min at 37°C, and then mounted for imaging using a fluorescent microscope (Olympus model BX51) fitted with DAPI (350 nm) FITC (488 nm) and TRITC (594 nm) filters and a DP70 camera adapter system (27).

**Table 1:** Target, host and source of primary and secondary antibodies employed for immunofluorescent islet histology and immunocytochemistry studies.

Primary antibodies				
Target	Host	Dilution		Source
Insulin	Mouse	1:500		Abcam, ab6995
Glucagon	Guinea pig	1:200		Raised in-house, PCA2/4
GFP	Goat	1:400		Abcam, ab5450
**Secondary antibodies**				
**Target**	**Host**	**Reactivity**	**Dilution**	**Fluorescent dye and source**
IgG	Goat	Mouse	1:400	Alexa Fluor^®^ 594, Invitrogen, UK
IgG	Goat	Guinea pig	1:400	Alexa Fluor^®^ 594, Invitrogen, UK
IgG	Goat	Guinea pig	1:400	Alexa Fluor^®^ 488, Invitrogen, UK
IgG	Donkey	Goat	1:400	Alexa Fluor^®^ 488, Invitrogen, UK

### Image Analysis

Cell^F^ imaging software (Olympus Soft Imaging Solutions) was used to analyze islet architectural parameters. Positive islet GFP staining was used to track lineage of alpha-cells in *Glu^CreERT2^*;*Rosa26-eYFP* mice and beta-cells in *Ins1^Cre/+^*;*Rosa26-eYFP* transgenic mice. For transdifferentiation, cells expressing GFP without insulin or glucagon were termed insulin^-ve^/GFP^+ve^ or glucagon^-ve^/GFP^+ve^, respectively. Cells co-expressing GFP with insulin or glucagon were termed insulin^+ve^/GFP^+ve^ or glucagon^+ve^/GFP^+ve^ cells, respectively. The proportion of insulin^+ve^/GFP^+ve^ or glucagon^+ve^/GFP^+ve^ cells was expressed as a percentage of the total number of GFP^+ve^ cells analyzed. Every cell that was positive for ether insulin, glucagon and/or GFP was counted. All cell counts were determined in a blinded manner with >100 islets analyzed per treatment group.

### Biochemical Analyses

Blood samples were collected from the cut tail vein of animals. Blood glucose was measured using a portable Ascencia Contour blood glucose meter (Bayer Healthcare, Newbury, Berkshire, UK). For plasma insulin and glucagon, blood was collected in chilled fluoride/heparin coated microcentrifuge tubes (Sarstedt, Numbrecht, Germany) and centrifuged using a Beckman micro-centrifuge (Beckman Instruments, Galway, Ireland) for 10 min at 12,000 rpm. Plasma was extracted and stored at −20°C, until required for analysis. For hormone content, snap frozen pancreatic tissues were homogenized in acid/ethanol (75% (v/v) ethanol, distilled water and 1.5% (v/v) 12 M HCl) and protein extracted in a pH neutral TRIS buffer. Protein content was determined using Bradford reagent (Sigma-Aldrich). Plasma and pancreatic insulin content were determined by an in-house insulin RIA (28), while plasma and pancreatic glucagon content were assessed by a commercially available ELISA kit (Glucagon chemiluminescent assay, EZGLU-30K, Millipore) following the manufacturer’s guidelines.

### Statistics

Data were analyzed using GraphPad PRISM 5.0, with data presented as mean ± SEM. Comparative analyses between control and experimental groups of *Glu^CreERT2^*;*Rosa26-eYFP* or *Ins1^Cre/+^*;*Rosa26-eYFP* mice were carried out using Student’s unpaired *t*-test, one-way ANOVA with a Bonferroni *post hoc* test or a two-way repeated measures ANOVA with a Bonferroni *post hoc* test, as appropriate. Results were deemed significant once p < 0.05.

## Results

### Effects of SL-PYY(1-36) on Body Weight and Cumulative Food and Fluid Intake in STZ-Diabetic Glu^CreERT2^;Rosa26-eYFP and Ins1^Cre/+^;Rosa26-eYFP Mice

**Figure 1:**
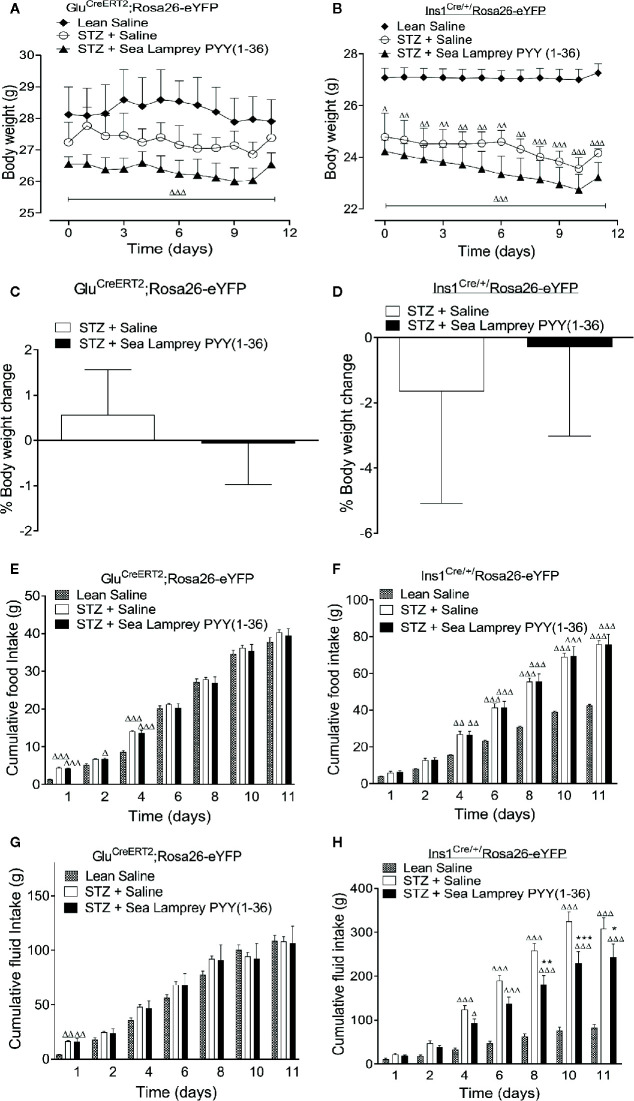
Effects of twice-daily administration of sea lamprey PYY [SL-PYY (1-36)] on **(A, B)** body weight, **(C, D)** % body weight change as well as cumulative **(E, F)** food and **(G, H)** fluid intake in streptozotocin (STZ)-diabetic *Glu^CreERT2^*;*Rosa26-eYFP* and *Ins1^Cre/+^*;*Rosa26-eYFP* mice. Parameters were measured prior to, and 11 days during, twice daily treatment with saline vehicle or SL-PYY(1-36) (25 nmol/kg bw) in STZ-diabetic *Glu^CreERT2^*;*Rosa26-eYFP* and *Ins1^Cre/+^*;*Rosa26-eYFP* mice. Values are mean ± SEM (n=6). ^Δ^p < 0.05, ^ΔΔ^p < 0.01, ^ΔΔΔ^p < 0.001 compared to appropriate non-diabetic control. *p < 0.05, **p < 0.01, ***p < 0.001 compared to appropriate STZ-diabetic control.

STZ induced a decline in body weight in *Glu^CreERT2^*;*Rosa26-eYFP* and *Ins1^Cre/+^*;*Rosa26-eYFP* mice (**Figures 1A, B**). Twice-daily SL-PYY(1-36) administration was unable to reverse this effect in either mouse model (**Figures 1A, B**), with overall percentage body weight change being remarkably similar to respective STZ-diabetic control mice (**Figures 1C, D**). There were differences between the mouse models in terms of food and fluid intake, with elevations (p < 0.001) of both parameters being only transient in nature in *Glu^CreERT2^*;*Rosa26-eYFP* STZ-diabetic mice and totally absent from day 4 onwards (**Figures 1E, G**). In *Ins1^Cre/+^*;*Rosa26-eYFP* mice, STZ elevated (p < 0.01 – p < 0.001) food and fluid intake from day 4 onwards (**Figures 1F, H**), with SL-PYY(1-36) significantly (p < 0.05 – p < 0.001) reducing increased fluid consumption on days 8, 10 and 11 (**Figure 1H**).

### Effects of SL-PYY(1-36) on Circulating Glucose, Insulin and Glucagon, as Well as Pancreatic Insulin and Glucagon Content, in STZ-Diabetic Glu^CreERT2^;Rosa26-eYFP and Ins1^Cre/+^;Rosa26-eYFP Mice

**Figure 2:**
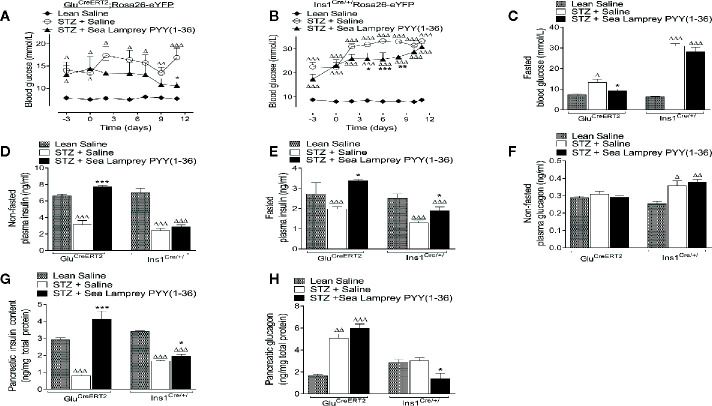
Effects of twice-daily administration of sea lamprey PYY [SL-PYY (1-36)] on blood glucose as well as plasma and pancreatic insulin and glucagon concentrations in streptozotocin (STZ)-diabetic *Glu^CreERT2^*;*Rosa26-eYFP* and *Ins1^Cre/+^*;*Rosa26-eYFP* mice. Blood glucose was measured at regular intervals during 11 days twice daily treatment with saline vehicle or SL-PYY(1-36) (25 nmol/kg bw) in STZ-diabetic **(A)** *Glu^CreERT2^*;*Rosa26-eYFP* or **(B)** *Ins1^Cre/+^*;*Rosa26-eYFP* mice. Terminal analysis included measurement of overnight fasted **(C)** glucose and **(E)** insulin, non-fasted **(D)** insulin, and **(F)** glucagon as well pancreatic **(G)** insulin and **(H)** glucagon. Values are mean ± SEM (n=6). ^Δ^p < 0.05, ^ΔΔ^p <.01, ^ΔΔΔ^p < 0.001 compared to appropriate non-diabetic control. *p < 0.05, **p < 0.01, ***p < 0.001 compared to appropriate STZ control.

Non-fasted circulating blood glucose levels were significantly (p < 0.05 – p < 0.001) elevated when compared to respective saline controls in STZ-diabetic *Glu^CreERT2^*;*Rosa26-eYFP* and *Ins1^Cre/+^*;*Rosa26-eYFP* mice (**Figures 2A, B**). SL-PYY(1-36) treatment reduced (p < 0.05 - p < 0.001) glucose concentrations in both mouse models, specifically, reversing and substantially delaying overt hyperglycaemia in *Glu^CreERT2^*;*Rosa26-eYFP* and *Ins1^Cre/+^*;*Rosa26-eYFP* mice, respectively (**Figures 2A, B**). Indeed, terminal blood glucose levels following a 16 hour fast were similar to non-diabetic controls in SL-PYY(1-36) treated *Glu^CreERT2^*;*Rosa26-eYFP* STZ mice, but still significantly (p < 0.001) elevated in *Ins1^Cre/+^*;*Rosa26-eYFP* treated STZ mice (**Figure 2C**). In agreement, corresponding fasted and non-fasted plasma insulin concentrations in SL-PYY(1-36) treated *Glu^CreERT2^*;*Rosa26-eYFP* animals were elevated (p < 0.001 and p < 0.05; respectively) compared to STZ-diabetic controls, and not different from non-diabetic control mice (**Figures 2D, E**). Insulin concentrations were also significantly reduced (p < 0.001) in all STZ-diabetic *Ins1^Cre/+^*;Rosa*26-eYFP* mice (**Figures 2D, E**). Interestingly, fasted insulin concentrations, but not non-fasted, were increased (p < 0.05) in SL-PYY(1-36) treated *Ins1^Cre/+^*;Rosa*26-eYFP* mice when compared to STZ-diabetic controls (**Figures 2D, E**), but still reduced (p < 0.001) in comparison to lean control mice (**Figure 2E**). Non-fasted plasma glucagon levels were not different in any of the *Glu^CreERT2^*;*Rosa26-eYFP* mice, but elevated (p < 0.05 and p < 0.01) when compared to non-diabetic controls in all STZ *Ins1^Cre/+^*;*Rosa26-eYFP* mice (**Figure 2F**). Pancreatic insulin content was dramatically (p < 0.001) increased in SL-PYY(1-36) treated *Glu^CreERT2^*;*Rosa26-eYFP* mice when compared to STZ-diabetic controls, with pancreatic glucagon elevated (p < 0.01 and p < 0.001) in all STZ *Glu^CreERT2^*;*Rosa26-eYFP* mice (**Figures 2G, H**). SL-PYY(1-36) treatment decreased (p < 0.05) pancreatic glucagon content when compared to STZ-diabetic and non-diabetic control *Ins1^Cre/+^*;*Rosa26-eYFP* mice (**Figure 2H**), with pancreatic insulin content increased (p < 0.05) compared to STZ-diabetic controls but still reduced (p < 0.001) in comparison to lean control mice (**Figure 2G**).

### Effects of SL-PYY(1-36) on Pancreatic Islet Morphology in STZ-Diabetic Glu^CreERT2^;Rosa26-eYFP and Ins1^Cre/+^;Rosa26-eYFP Mice

**Figure 3:**
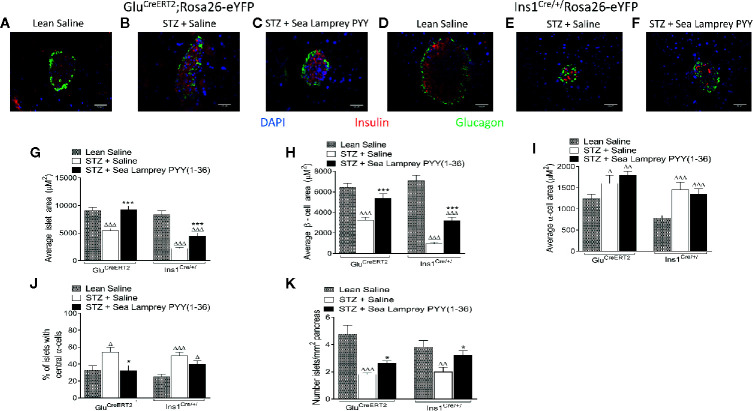
Effects of twice-daily administration of sea lamprey PYY [SL-PYY (1-36)] on pancreatic islet morphology in streptozotocin (STZ)-diabetic *Glu^CreERT2^*;*Rosa26-eYFP* and *Ins1^Cre/+^*;*Rosa26-eYFP* mice. Parameters were assessed after 11 days treatment with saline vehicle or SL-PYY(1-36) (25 nmol/kg bw). **(A–F)** Representative images (40×) from each treatment group for **(A–C)** *Glu^CreERT2^*;*Rosa26-eYFP* and **(D–F)** *Ins1* ^Cre/+^;*Rosa26-eYFP* mice are depicted. Average **(D)** islet, **(H)** beta-, and **(I)** alpha-cell areas, as well as **(J)** the proportion of islets possessing centrally located alpha-cells and **(K)** number of islets per mm^2^. Values are means ± SEM of 6 mice per group, with approximately 100 islets being analyzed per group. ^Δ^p < 0.05, ^ΔΔ^p < 0.01, ^ΔΔΔ^p < 0.001 compared to appropriate non-diabetic control. *p < 0.05, ***p < 0.001 compared to appropriate STZ control.

The detrimental effects of multiple low dose STZ administration on pancreatic islet morphology in both *Glu^CreERT2^*;*Rosa26-eYFP* and *Ins1^Cre/+^*;*Rosa26-eYFP* mice is clear from the representative islet images depicted in **Figures 3A–F**, and largely consistent between models apart from substantially greater beta-cell loss in *Ins1^Cre/+^*;*Rosa26-eYFP* mice. Specifically, STZ induced significant (p < 0.001) reductions in average islet and beta-cell areas, with increased (p < 0.05 to p < 0.001) alpha-cell area (**Figures 3G–I**) in both transgenic mouse models. SL-PYY(1-36) had no impact on STZ-induced elevations of pancreatic alpha-cell area (**Figure 3I**). In contrast, SL-PYY(1-36) treatment substantially (p < 0.001) increased islet and beta-cell areas in both *Glu^CreERT2^*;*Rosa26-eYFP* and *Ins1^Cre/+^*;*Rosa26-eYFP* mice (**Figures 3G, H**). Indeed, these parameters were similar to non-diabetic control mice in the *Glu^CreERT2^*;*Rosa26-eYFP* model (**Figures 3G, H**). Interestingly, SL-PYY(1-36)-induced changes of islet cell areas in both models were accompanied by architectural modifications that included a reduced penetration of alpha-cells into the central portion of the islet, especially in *Glu^CreERT2^*;*Rosa26-eYFP* animals (**Figure 3J**) and increased (p < 0.05) numbers of islets per mm^2^ tissue (**Figure 3K**).

### Effects of SL-PYY(1-36) on Islet Cell Lineage in STZ-Diabetic Glu^CreERT2^;Rosa26-eYFP and Ins1^Cre/+^;Rosa26-eYFP Mice

**Figure 4:**
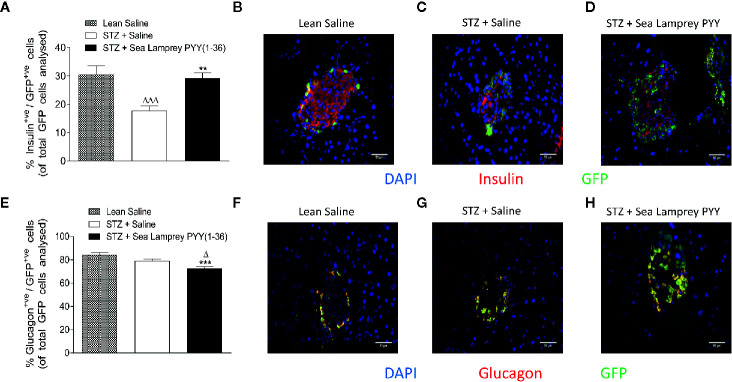
Effects of twice-daily administration of sea lamprey PYY [SL-PYY (1-36)] on islet cell lineage in streptozotocin (STZ)-diabetic *Glu^CreERT2^*;*Rosa26-eYFP* mice. Parameters were assessed after 11 days treatment with saline vehicle or SL-PYY(1-36) (25 nmol/kg bw). Quantification of **(A)** glucagon^+ve^/GFP^+ve^ and **(E)** insulin^+ve^/GFP^+ve^ islet stained cells. Representative images (40×) of islets showing **(B–D)** insulin (red) or **(F–H)** glucagon (red) as well as GFP (green) and DAPI (blue) immunoreactivity from each group of mice; scale bar 50 μm. Values are means ± SEM of six mice per group, with approximately 100 islets being analyzed per group. ^Δ^p < 0.05, ^ΔΔΔ^p < 0.001 compared to appropriate non-diabetic control. **p < 0.01, ***p < 0.001 compared to appropriate STZ control.

**Figure 5:**
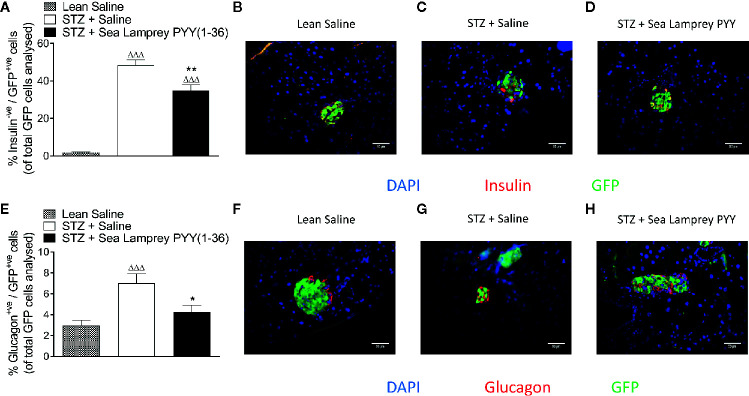
Effects of twice-daily administration of sea lamprey PYY [SL-PYY(1–36)] on islet cell lineage in streptozotocin (STZ)-diabetic *Ins1^Cre/+^*;*Rosa26-eYFP* mice. Parameters were assessed after 11 days treatment with saline vehicle or SL-PYY(1–36) (25 nmol/kg bw). Quantification of **(A)** insulin^-ve^/GFP^+ve^ and **(E)** glucagon^+ve^/GFP^+ve^ islet stained cells. Representative images (40×) of islets showing **(B–D)** insulin (red) or **(F–H)** glucagon (red) as well as GFP (green) and DAPI (blue) immunoreactivity from each group of mice; scale bar 50 μm. Values are means ± SEM of six mice per group, with approximately 100 islets being analyzed per group. ^ΔΔΔ^p < 0.001 compared to appropriate non-diabetic control. *p < 0.05, **p < 0.01 compared to appropriate STZ control.

In *Glu^CreERT2^*;*Rosa26-eYFP* mice, STZ resulted in a reduction (p < 0.001) in the number of cells co-expressing insulin and GFP (insulin^+ve^/GFP^+ve^), an effect that was fully reversed (p < 0.01) by twice daily SL-PYY(1-36) treatment (**Figure 4A**). Representative images of islets from Glu^CreERT2^;*Rosa26-eYFP* and Ins1^Cre/+^ *;Rosa26-eYFP* mice co-stained for GFP and insulin or glucagon are shown in **Figures 5B–D, F–H**. Representative images of insulin and GFP stained islets from each group of mice are provided in **Figures 4B–D**. Interestingly, while STZ did not alter the number of islet cells co-expressing glucagon and GFP (glucagon^+ve^/GFP^+ve^) in *Glu^CreERT2^*;*Rosa26-eYFP* mice, SL-PYY(1-36) decreased the number of these co-expressing cells when compared to both STZ-diabetic (p < 0.001) and non-diabetic (p < 0.05) control mice (**Figures 4E**). Representative images of glucagon and GFP stained islets are depicted in **Figures 4F–H**. Similar, albeit in mice with non-inducible GFP expression, islet cell lineage investigations were conducted in *Ins1^Cre/+^*;*Rosa26-eYFP* mice, revealing comparable beneficial effects of SL-PYY(1-36) despite differences in the diabetes phenotype of the two transgenic models (**Figure 5**). As such, STZ treatment in this mouse model resulted in increased (p < 0.001) percentages of insulin negative, GFP positive (insulin^-ve^/GFP^+ve^) as well as glucagon^+ve^/GFP^+ve^ cells (**Figures 5A, E**). Twice daily SL-PYY(1-36) treatment significantly (p < 0.01) reduced percentages of insulin^-ve^/GFP^+ve^ islet cells (**Figure 5A**) and returned percentages of glucagon^+ve^/GFP^+ve^ cells similar to those observed in non-diabetic control mice (**Figures 5E**). Representative images of islets from *Glu^CreERT2^*;*Rosa26-eYFP* and *Ins1^Cre/+^*;*Rosa26-eYFP* mice co-stained for GFP and insulin or glucagon are shown in **Figures 5B–D, F–H**.

## Discussion

As expected, multiple low dose STZ administration resulted in characteristic pancreatic islet beta-cell destruction, acute insulin deficiency and elevations of blood glucose in both *Glu^CreERT2^*;*Rosa26-eYFP* and *Ins1^Cre/+^*;*Rosa26-eYFP* transgenic mice. Interestingly, appreciable differences were apparent between these transgenic mice in terms of STZ susceptibility and effects on body weight, food and fluid intake as well as alpha-cell derived circulating glucagon and degree of hyperglycaemia, that indicated a more severe diabetic phenotype in *Ins1^Cre/+^*;*Rosa26-eYFP* mice.

Although unlikely given the mechanism of action of STZ (29), presence of the transgene within beta-cells of *Ins1^Cre/+^*;*Rosa26-eYFP* mice could contribute to the difference in susceptibility to the toxin. However, studies in *Glu^CreERT2^*;*ROSA26-eYF*P mice confirm that activation of the Cre recombinase enzyme does not affect normal islet cell processes (22), and although unlikely, production of fluorescent GFP protein within beta cells of *Ins1^Cre/+^*;*Rosa26-eYFP* mice could increase metabolic demand leaving cells more susceptible to STZ. *Glu^CreERT2^*;*Rosa26-eYFP* mice, unlike *Ins1^Cre/+^*;*Rosa26-eYFP*, also require a single, low-dose, tamoxifen injection to induce Cre-lox recombination, raising the possibility that estrogen receptor antagonism might curb some detrimental effects of STZ, but this appears only to occur during sub-chronic dosing regimen (30). Indeed, the use of tamoxifen-dependent Cre recombinase induction is a commonly employed tool to successfully study gene function with minimal adverse effects. However, we recognize that an additional group of control *Glu^CreERT2^*;*Rosa26-eYFP* mice would have been useful to confirm lack of effect of the single dose of tamoxifen on diabetic phenotype. A more likely explanation relates to variations in STZ susceptibility between (25), and even within (26), strains of mice, comparable to the established gender variations of STZ effects in rodents (31). However, given both transgenic mouse models displayed clear STZ-induced beta-cell ablation, with distinct diabetes-induced alterations in islet cell transitioning (23, 32), they represent valid tools to assess the impact of sustained NPY1R agonism on alpha- to beta- and beta- to alpha-cell transdifferentiation. Moreover, the difference in presenting diabetic phenotype of *Glu^CreERT2^*;*Rosa26-eYFP* and *Ins1^Cre/+^*;*Rosa26-eYFP* mice allows for differentiation between direct positive effects of SL-PYY(1-36) on islet cell transdifferentiation, as opposed to indirect benefits related to improved glycaemic control and metabolic status. However, because *Glu^CreERT2^* and *Ins1^Cre/+^* are not equivalent lineage tracers, labeling islet cells either during tamoxifen exposure or from the moment of *Ins1* expression, respectively, direct quantitative comparisons of islet cell changes between the models should be avoided.

In keeping with earlier observations (4), SL-PYY(1-36) increased beta-cell area, plasma and pancreatic insulin in both *Glu^CreERT2^*;*Rosa26-eYFP* and *Ins1^Cre/+^*;*Rosa26-eYFP* mice, as well as reversing the characteristic infiltration of glucagon positively stained central alpha-like cells (4, 5). Beta-cell benefits are undoubtedly linked, in part, to increased proliferation and reduced beta-cell apoptosis, as documented previously with sustained NPY1R activation in diabetic mouse models (1, 4, 5). Interestingly, previous studies in insulin-deficient mice also demonstrated reductions in alpha-cell area following SL-PYY(1-36) treatment, which coupled with increased beta-cell mass, resulted in no obvious change in overall pancreatic islet area (4, 5). In the current setting, islet area increased with 11-day SL-PYY(1-36) treatment in both diabetic transgenic models, as a result of elevations in alpha- and beta-cell areas. We assume that the slightly different scenario compared to our previous study (4) is related to differences in the strain of mice employed and associated physiological responses to STZ (26), as well as duration of SL-PYY(1-36) treatment. Nonetheless, observed metabolic and islet benefits reaffirm the recognized beta-cell pro-survival effects of NPYR1 signaling (16). This established benefit, coupled with evidence of beta-cell loss following selective KO of PYY expressing cells (1), as well as expansion of PYY expressing alpha-cell like islet cell populations during regeneration of the adult pancreas (33), indicate the importance of NPY receptor signaling for positive islet cell adaptations in response to metabolic stress. Furthermore, upregulation of NPY receptor activation has also been linked to the improved insulin secretion and resolution of diabetes following bariatric surgery (34–36).

In terms of islet cell lineage tracing, studies in *Ins1^Cre/+^*;*Rosa26-eYFP* mice revealed that STZ induced notable increases in the number of pancreatic beta-cells losing their identity, as well as mature beta-cells transitioning to glucagon positive alpha-like cells (23). Interestingly, in *Glu^CreERT2^*;*Rosa26-eYFP* mice a seemingly high intrinsic transition of glucagon positive alpha-cells to insulin positive beta-cells was decreased by STZ intervention. Triple staining of GFP, glucagon and insulin would have been useful to provide additional insight on these islet cell lineage processes, especially in *Glu^CreERT2^*;*Rosa26-eYFP* mice, but was not possible due to methodological constraints. Furthermore, STZ susceptibility of cells transitioning from/to beta-cells and the relevance of established cytoprotective effects of SL-PYY(1-36) in such cell populations are unknown. However, when taken together, the data suggest that a likely source of the additional positively stained glucagon cells in both diabetic models in the present study is linked to transdifferentiation of beta-cells toward alpha-cells and possible lack of alpha-cell dedifferentiation, although the latter point requires further investigation. Most importantly, SL-PYY(1-36) treatment appeared to be able to fully, or partially, counter each of these STZ-induced detrimental islet cell transdifferentiation events. Intriguingly, despite increased alpha-cell area, pancreatic glucagon content was decreased by SL-PYY(1-36) treatment in both mouse models. This might suggest that a significant number of alpha-cells were currently in the process of transition, probably toward a more beta-cell like phenotype, and therefore have reduced glucagon content. In full agreement, SL-PYY(1-36) reduced the percentage of glucagon^+ve^/GFP^+ve^ cells in *Glu^CreERT2^*;*Rosa26-eYFP* mice, and augmented alpha- to beta-cell transdifferentiation. The location on different mouse islet cells of NPY1R, which we detected previously to be expressed at relatively low levels by alpha-, beta- and delta-cells and the role of endogenous PYY, expressed in adult mouse alpha- and delta-cells (37), will need to be determined in the future. In terms of alpha-cell secretion, non-fasted circulating glucagon concentrations were unaltered by SL-PYY(1-36), however it is envisaged that any effect on glucagon would be more apparent under fasting conditions, which unfortunately were not assessed in the current study.

Unlike *Ins1^Cre/+^*;Rosa*26-eYFP* mice, the *Glu^CreERT2^*;*Rosa26-eYFP* transgenic model exhibited an almost complete restoration of normoglycaemia, suggesting that exogenous peptide administration can directly influence islet cell transdifferentiation. As such, improvements in glycaemic status have been shown to independently alter pancreatic islet cell lineage (38). Related NPYR1 dependent mechanisms still need to be investigated, and although beyond the scope of the current study, consideration of levels of specific alpha- and beta-cell transcription factors such as aristaless-related homeobox (Arx) (39), paired box gene 4 (Pax4) (40), pancreatic and duodenal homeobox 1 (Pdx-1) or forkhead box O1 (FOXO1) (41) would be interesting. Indeed, in the absence of such additional investigations, some constraint is required in terms of interpretation of the full impact of SL-PYY(1-36) on transdifferentiation of alpha- to beta-cells. Findings should also be interpreted in relation to the decrease in islet PYY expression observed following STZ-induced insulin deficiency in rodents (1, 16), although islet PYY levels were not directly assessed in the current setting. In addition, observations of increased numbers of individual islets following SL-PYY(1-36) treatment might suggest islet neogenesis is involved in beneficial NPYR1 mediated islet adaptations, but this would also require further, more detailed study. Indeed, an established role for PYY during rapid islet cell development and replication within the embryo makes this scenario plausible and worthy of investigation (42). In this regard, it may be interesting to assess the impact of SL-PYY(1-36) on islet cell lineage under normal physiological conditions.

## Conclusion

The present study demonstrates that sustained NPYR1 activation by SL-PYY(1-36) rescued beta-cell loss and augmented beta-cell function in chemically induced insulin-deficient diabetes, leading to significantly enhanced overall metabolic control. As well as increased proliferation and reduced apoptosis of beta-cells (3–5, 16), SL-PYY(1-36) also appeared to positively influence the transitioning of both islet alpha- and beta-cells. This newly discovered, beneficial islet cell lineage effect emphasizes the potential of stable long-acting NPYR1 agonists to promote prevailing, disease-modifying benefits in diabetes, linked to possible alteration of alpha cell function and transdifferentiation of alpha- to beta-cells.

## Data Availability Statement

The raw data supporting the conclusions of this article will be made available by the authors, without undue reservation.

## Ethics Statement

The animal study was reviewed and approved by the Ulster University Animal Welfare and Ethical Review Body (AWERB).

## Author Contributions

RL and NT performed the experiments and prepared the first draft of the manuscript. RM, PF, and NI designed the experiments and reviewed and revised all subsequent versions of the manuscript. FR and FG reviewed and revised the manuscript. All authors contributed to the article and approved the submitted version.

## Funding

This work was supported by a PhD studentship (awarded to RL) from the Department for the Economy (DfE) Northern Ireland and University of Ulster strategic research funding. Work in the Reimann/Gribble laboratory is currently funded by the Welcome Trust (106262/Z/14/Z and 106263/Z/14/Z) and the MRC (MRC_MC_UU_12012/3).

## Conflict of Interest

PF and NI are named on patents filed by the University of Ulster for exploitation of peptide therapeutics.

The remaining authors declare that the research was conducted in the absence of any commercial or financial relationships that could be construed as a potential conflict of interest.
